# Proportional Tumor Infiltration of T Cells *via* Circulation Duplicates the T Cell Receptor Repertoire in a Bilateral Tumor Mouse Model

**DOI:** 10.3389/fimmu.2021.744381

**Published:** 2021-10-25

**Authors:** Mikiya Tsunoda, Hiroyasu Aoki, Haruka Shimizu, Shigeyuki Shichino, Kouji Matsushima, Satoshi Ueha

**Affiliations:** ^1^ Division of Molecular Regulation of Inflammatory and Immune Diseases, Research Institute for Biomedical Sciences, Tokyo University of Science, Chiba, Japan; ^2^ Department of Medicinal and Life Sciences, Faculty of Pharmaceutical Sciences, Tokyo University of Science, Chiba, Japan; ^3^ Department of Hygiene, Graduate School of Medicine, The University of Tokyo, Tokyo, Japan

**Keywords:** cancer immunotherapy, immunomonitoring, TCR sequencing, overlap analysis, TCR repertoire

## Abstract

Temporal analysis of the T cell receptor (TCR) repertoire has been used to monitor treatment-induced changes in antigen-specific T cells in patients with cancer. However, the lack of experimental models that allow a temporal analysis of the TCR repertoire in the same individual in a homogeneous population limits the understanding of the causal relationship between changes in TCR repertoire and antitumor responses. A bilateral tumor model, where tumor cells were inoculated bilaterally into the backs of mice, could be used for temporal analysis of the TCR repertoire. This study examined the prerequisite for this strategy: the TCR repertoire is conserved between bilateral tumors that grow symmetrically. Bilateral tumors and draining lymph nodes (dLNs) were collected 13 days after tumor inoculation to analyze the TCR repertoire of CD4^+^ and CD8^+^ T cells. The tumor-infiltrating T-cell clones were highly similar between the bilateral tumors and expanded to a similar extent. In addition, the differences of TCR repertoire between the bilateral tumors were equivalent to Intra-tumoral heterogeneity on one side. On the other hand, the similarity of the TCR repertoire in the bilateral dLNs was markedly lower than that in the tumor, suggesting that tumor-reactive T cell clones induced independently in each dLN are mixed during recirculation and then proportionally infiltrated the bilateral tumors. These findings provide the basis for future analysis of temporal and treatment-induced changes in tumor-reactive T cell clones using this bilateral tumor model.

## Introduction

Immune checkpoint inhibitors (ICIs) have a significant therapeutic effect in some cancers and have become an important pillar of cancer treatment in recent years (1, 2). However, the response rate to ICI monotherapy is less than 30% for most types of cancer, and ICIs occasionally cause severe immune-related adverse effects in some patients (3, 4). Thus, the development of reliable biomarkers that represent tumor-specific immune responses and stratify the responder and non-responder at an early stage is essential to optimize the usage of ICIs (5, 6).

Because ICIs suppresses tumor growth by enhancing the proliferation and activation of tumor-specific T cells (7), the efficacy of ICIs is closely associated with the strength of tumor-specific T cell responses. These tumor-specific T cells are composed of various tumor-reactive T cell clones with different specificities to tumor-associated antigens (8, 9). Antigen specificity of T cell clones is determined by their T cell receptors (TCRs), which are generated by V(D)J recombination in the thymus and are incredibly diverse (10, 11). Therefore, global analysis of the collection of TCRs using next-generation sequencing, that is TCR repertoire analysis, can now be applied to monitor tumor-specific T cell responses in patients receiving ICIs (12, 13).

Several studies have reported the diagnostic or predictive features of the TCR repertoire in mice treated with immunotherapy. Philip (14) and Rudqvist (15) reported that mice treated with an anti-cytotoxic T lymphocyte-associated protein 4 monoclonal antibody had a more clonal TCR repertoire of tumor-infiltrating T cells. We also reported that mice deprived of CD4^+^ immunosuppressive cells had an increase in the number and frequency of CD8^+^ T cell clones that are detected throughout the tumor, draining lymph node (dLN), and peripheral blood (16). Based on the cancer-immunity cycle, where tumor-specific T cells are primed in the dLN and infiltrate into the tumor *via* lymph–blood circulation (17), these “overlapping” T cell clones seem to reflect the mobilization of T cells into antitumor responses. Consistent with this hypothesis, the diversity and total frequency of these overlapping CD8^+^ T cell clones were associated with antitumor effects in mice and humans (16, 18). The findings in mice have revealed the diagnostic or predictive features of the TCR repertoire in individuals who achieved antitumor responses. However, it remained unclear whether the immunotherapy-associated features of the TCR repertoire were a cause or a result of anti-tumor T cell responses. To elucidate the causal relationship between TCR repertoire and antitumor response, temporal monitoring of tumor T-cell repertoire in individual mice is required. However, it is difficult to prepare a sufficient amount of biopsy samples from the tiny mouse tumors without affecting tumor growth.

Recently, Zemek (19) and Chen (20) reported that the immune microenvironment is similar in bilateral tumor models with comparable tumor growth and that it can be applied to the temporal analysis of antitumor immune responses. To apply this method for temporal analysis of the TCR repertoire, there are important prerequisites: (1) the TCR repertoires of the left and right tumors show similar characteristics; and (2) the same tumor-reactive T cell clones infiltrate into the bilateral tumors. In this study, we investigated whether the bilateral tumor model is suitable for examining the temporal responses of tumor-reactive T cells in individual mice.

## Materials and Methods

### Mice and Cell Line

Eight-week-old female C57BL/6J mice were purchased from Sankyo Labo service corporation inc. (Tokyo, Japan). Lewis lung carcinoma (LLC) was originally provided from the Nihonkayaku (Tokyo, Japan).

### Tumor Inoculation

LLC cells (5 × 10^5^ cells) were inoculated subcutaneously (s.c.) into the bilateral backs of C57BL/6J mice. Tumor diameter was measured twice a week and tumor volume (mm^3^) was calculated according to the following formula [(major axis; mm) x (minor axis; mm)^2^ x 0.5]. In some mice, 1% Evans Blue dye (Sigma-Aldrich, Tokyo, Japan) was injected into the tumor 30 min prior to sacrifice to determine the draining lymph node (dLN) in our model. All animal experiments were conducted in accordance with institutional guidelines with the approval of the Animal Care and Use Committee of the Tokyo University of Science.

### Flow Cytometry and Cell Sorting

Intravascular leukocytes were stained by intravenous injection of FITC-conjugated monoclonal antibody (mAb) (3 µg/mouse) against CD45.2 (clone 104) three minutes before sacrifice (21). The Tumor was equally divided into two parts and processed individually. Each tumor was cut into small fragments and digested for 45 minutes at 37°C with 0.1% collagenase (032-22364, FUJIFILM Wako, Osaka, Japan). The cells were then subjected to density separation with 40% Percoll PLUS (Cytiva, Marlborough, MA) and leukocytes were recovered from the bottom layer. Ammonium Chloride Potassium (ACK) Lysing buffer was used to lyse red blood cells. The extracted dLN was cut into small fragments and mashed on a cell strainer. The cell number was determined using Flow-Count fluorospheres (Beckman Coulter, San Diego, CA) and a CytoFLEX flow cytometer (Beckman Coulter). Cells were then stained with a mix of Fc Block (anti-mouse CD16/CD32 mAb; clone 2.4G2, BioXcell) and fluorophore-conjugated anti-mouse mAbs as indicated in **Supplementary Table S1**. After enrichment of T cells with magnetic separation by Dynabeads M-280 Streptavidin (Thermo Fisher Scientific, Tokyo, Japan), CD8^+^ and CD4^+^ T cells from the tumor and CD8^+^ CD44^hi^ and CD4^+^ CD44^hi^ T cells from the dLN were sorted using FACS Aria II or Aria III cell sorter (BD Biosciences, San Jose, CA) The number of CD8^+^ and CD4^+^ T cells sorted from each tumor and dLN was shown in **Supplementary Table S2**. Propidium iodide-positive cells were excluded as nonviable cells, and intravascular staining-CD45.2 positive cells were also excluded as non-tumor-infiltrated cells. The purity of sorted cells was always over 95%. Data were analyzed using FlowJo software (version 10.5.3; BD Biosciences).

### TCR Library Construction and Sequencing

TCR libraries were prepared on purified T cells lysed in lysis buffer [1% Lithium Lauryl sulfate (NACALAI TESQUE, Kyoto, Japan), 100 mM Tris-HCl (pH 7.5) (NIPPON GENE, Tokyo, Japan), 500 mM LiCl (Sigma-Aldrich), and 10 mM EDTA (NIPPON GENE)]. PolyA RNAs were isolated according to a previous report with some modifications (GSE110711). To perform reverse transcription and template-switching, mRNA-trapped oligo-dT-immobilized Dynabeads M270-streptavidin (Thermo Fisher Scientific) were suspended in 10 µL of RT mix [1× First Strand buffer (Thermo Fisher Scientific), 1 mM dNTP, 2.5 mM DTT (Thermo Fisher Scientific), 1 M betaine (Sigma-Aldrich), 9 mM MgCl2 (NIPPON GENE), 1 U/µL RNaseIn Plus RNase Inhibitor (Promega, Madison, WI), 10 U/µL Superscript II (Thermo Fisher Scientific), and 1 µM of i5-TSO], and incubated for 60 min at 42°C and immediately cooled on ice. Beads were washed once with B&W-T buffer [5 mM Tris-HCl (pH 7.5), 1 M NaCl (NACALAI TESQUE), 0.5 mM EDTA, and 0.1% Tween-20 (Sigma-Aldrich)], and once with Tris-HCl (pH 8.0). To amplify the TCR cDNA containing complementarity determining region 3 (CDR3), nested PCR of the TCR locus was performed as follows. cDNA-immobilized beads were resuspended with the 25 µL of first PCR mixture [0.4 μM of primers (i5, Trac_ex, and Trbc_ex), and 1x KAPA Hifi Hotstart ReadyMix (KAPA Biosystems, Wilmington, MA)], and the thermal cycling was performed as the following condition: denaturation at 95°C for 3 min, 5 cycles of denaturation for 20 sec at 98°C, annealing for 15 sec at 65°C and extension for 30 sec at 72°C, followed by a final extension at 72°C for 2 min. Then, 2.5µL of first PCR product was mixed with the 22.5 μL of second PCR mixture [0.35 μM of primers (i5_2nd and i7-BC_mTrbc), and 1x KAPA Hifi Hotstart ReadyMix], and the thermal cycling was performed under the same condition as first PCR. The second-PCR products were purified by an Agencort AM Pure XP kit (Beckman Coulter, CA) at a 0.7:1 ratio of beads to sample and eluted with 20 μL of 10 mM Tris-HCl (pH 8.0). To amplify TCR libraries and add adaptor sequences for the next-generation sequencer, the third PCR was performed as follows. 5 µL of purified second PCR product was mixed with the 20 μL of third PCR mixture [0.4 μM of primers (i5-BC and i7-BC), and 1x KAPA Hifi Hotstart ReadyMix], and the thermal cycling was performed under the same condition as first PCR, excepted for the number of cycles (23 cycles). The third-PCR products were purified as second PCR. The products were pooled and then purified and subjected to dual size selection using ProNex size-selective purification system (Promega) and eluted with 25 μL of 10 mM Tris-HCl (pH 8.5). Final TCR libraries, whose lengths were about 600 base pairs were sequenced using an Illumina Novaseq 6000 S4 flowcell (67 bp read 1 and 140 bp read 2) (Illumina, USA). Only read2 contained the sequence regarding the definition of T cell clones.

### Data Processing of TCR Sequencing

Adapter trimming and quality filtering of sequencing data were performed using Cutadapt-3.2 (22) and PRINSEQ-0.20.4 (23). Sequencing data were processed by MiXCR-3.0.5 (24). In MiXCR, Filtered reads were aligned to reference mouse TCR V/D/J sequences registered in the international ImMunoGeneTics information system with the following parameters: -starting-material=rna, -5-end=no-v-primers-, -3-end=c-primers, -adapters=no-adapters, vParameters.geneFeatureToAlign=VTranscript, -vjAlignmentOrder=JThenV. Then, identical sequences were assembled and grouped in clones with PCR and sequencing error correlation with the following parameters: -badQualityThreshold=15, –separateByV=true, –separateByJ=true, -only-productive=true, –region-of-interest=CDR3. The Variable (V) and Joining (J) segment of TCRs were represented in IMGT gene nomenclature. The list of final clones was analyzed by VDJtools-1.2.1 (25). Sequencing reads of the sample were normalized to six times of cell count in each sample by the “DownSample” command of VDJtools. T cell clones were determined as TCR reads with the same TCR V segment, J segment, and CDR3 nucleotide sequence. After normalization, the frequency of the clone was calculated as the number of reads for a particular clone divided by the total number of reads for all clones in the repertoire. The number of total reads and unique clones for each sample were shown in **Supplementary Table S2**. TCR repertoires of divided tumors were pooled other than **Figure 3**. The processed data have been deposited in NCBI GEO under the accession GSE174225.

### Analysis of the Indices of TCR Repertoire and the Extent of Overlap Between Repertoires

V/J segment usage plots of the TCR repertoire of bilateral tumors were generated by the “PlotFancyVJUsage” command of VDJtools. Principle component analysis of V and J segment usage was performed based on the frequency of each V or J segment using the prcomp function of R (version 3.6.0). The 1 - Pielou index was used to evaluate the clonality of TCR repertoire, which was calculated using the formula: $1-\Sigma_{i=1}^{n}p_{i}\log_{e}(p_{i})/\log_{e}(n)$ where *p_i_* is the frequency of cloning *i* for a sample with n unique clones. The Morisita-Horn index was used to estimate the similarity of TCR repertoire between bilateral tumors, which was calculated using the formula:


$$C_{H}=\frac{2\;\Sigma_{i=1}^{S}x_{i}y_{i}}{\left(\frac{\Sigma_{i=1}^{S}x_{i}^{2}}{X^{2}}+\frac{\Sigma_{i=1}^{S}y_{i}^{2}}{Y^{2}}\right)XY}$$

where *X_i_* is the number of clones *_i_* in the total X reads of one sample, *y_i_* is the number of clones *_i_* in the total Y reads of another sample, and S is the number of clones.

The frequency of overlap (OL) clones between samples is calculated by the geometric mean of the frequencies within each sample. The scatter plot in **Figures 7**, **8** was depicted using the ggplot2 package (26). The heatmap in **Supplementary Figures 2, 4, 7** was depicted using the complexheatmap package (27).

### Identification of Differentially Expanded Clones

Clones that differentially expanded in one side of the tumor (differentially expanded clones) were defined in Dewitt et al., using Fisher exact test on an estimated cell count of T cell clones, including clones detected only at one-time point (28). The estimated cell count was obtained by multiplying the T cell count for library preparation and the frequency of each clone, and q values corresponding to the p values of Fisher exact test were calculated using the qvalue package in Microsoft R open 3.6.0. We adopted q < 0.01 and fold change of clone’s frequency > 2 for the threshold of differentially expanded clones.

### Statistical Analysis

Statistical analyses were performed using GraphPad Prism software (version 8, GraphPad Software, La Jolla, CA). A two-sided paired Student’s t-test was run on the comparison of the frequency of dLN-Tumor overlapping clones between ipsilateral and contralateral ones. Ordinary one-way analysis of variance was run to compare the clonality of TCR repertoires between bilateral tumors and between individuals. All other experimental data were analyzed using a two-sided unpaired Student’s t-test. Asterisks to indicate significance corresponding to the following: n.s., not significant (P > 0.05), *P ≤ 0.05, **P ≤ 0.01, ***P ≤ 0.001, ****P ≤ 0.0001.

## Results

### TCR Repertoire of Bilateral Tumors Exhibits Similar Characteristics

**Figure 1:**
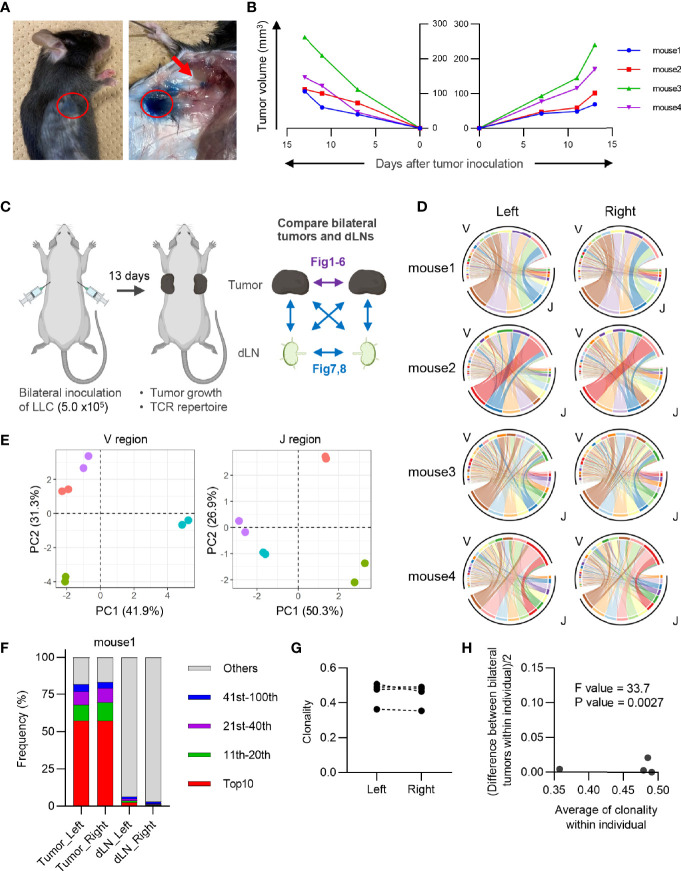
Characteristics of CD8^+^ T cell repertoire in the bilateral tumor. **(A)**, Visualization of draining lymph node (dLN) in our bilateral tumor model. 1% Evans Blue dye was injected into the tumor (red circle) 30 min prior to euthanasia. Brachial LN (arrow) was stained by Evans Blue, indicating this LN became a dLN. **(B)**, Growth curve of the tumor inoculated bilaterally on the backs. Lines of the same color indicate left and right tumors of the same mouse (n = 4). **(C)**, Experimental scheme for the entire study. Results of the comparison between bilateral tumors are presented in **Figures 1**–**6**, and those between bilateral dLNs or dLN and tumor are presented in **Figures 7**, **8**. **(D)**, V/J segment usage plots of the bilateral CD8^+^ T cell repertoires. Ribbons connecting the V and J segments are scaled by the corresponding V/J pair frequency. The color of the circumference represents the same V and J genes, and the color of the ribbon represents the same J genes (n = 4). **(E)**, Principal component analysis of V and J segment usage of tumor-infiltrating CD8^+^ T cells. Dots of the same color indicate left and right tumors of the same individual (n = 4). **(F)**, Frequency of abundant CD8^+^ T cell clones in the tumor and dLN. CD8^+^ T cell clones were categorized into five classes based on their rank in each repertoire: top 10, 11^th^–20^th^, 21^st^–40^th^, 41^st^–100^th^, and others. The total frequency of clones in each class is shown (n = 4). **(G)**, Clonality of the CD8^+^ T cell repertoire of the left and right tumor (n = 4). **(H)**, Homoscedasticity plot for variance of clonality in individual mice. The X-axis represents the average clonality of bilateral tumors in each individual (n = 4). The Y-axis represents variance of clonality between different bilateral tumors within individuals. Sum of squares within mouse = 9.7 × 10^-4^; sum of squares between mice = 2.4 × 10^-2^; degree of freedom within mouse = 4; degree of freedom between mice = 3.

We used the bilateral tumor model to establish an experimental system for evaluating temporal and treatment-induced changes in tumor-reactive T cell clones. To this end, the growth rate and clonal T cell responses in bilateral tumors must be similar in individual mice. Therefore, we first examined whether the Lewis lung carcinoma (LLC) tumors inoculated bilaterally into the back of individual mice grew symmetrically. Intratumoral injection of Evans Blue verified that the brachial lymph node became a dLN in the subcutaneous tumors (**Figure 1A**). The growth curves of bilateral tumors were symmetrical in individual mice, suggesting that a similar antitumor response occurred on both sides of the tumor (**Figure 1B**).

**Figure 2:**
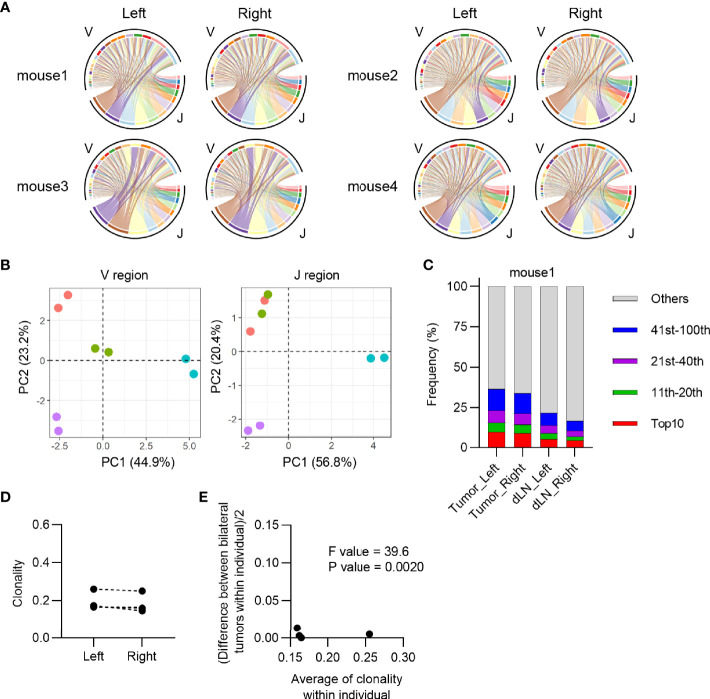
Characteristics of CD4^+^ T cell repertoire in the bilateral tumor. **(A)**, V/J segment usage plots of the bilateral CD4^+^ T cell repertoires. Ribbons connecting the V and J segments are scaled by the corresponding V/J pair frequency. The color of the circumference represents the same V and J genes, and the color of the ribbon represents the same J genes (n = 4). **(B)**, Principal component analysis of V and J segment usage of tumor-infiltrating CD4^+^ T cells. Dots of the same color indicate left and right tumors of the same individual (n = 4). **(C)**, Frequency of abundant CD4^+^ T cell clones in the tumor and dLN. CD4^+^ T cell clones were categorized into five classes based on their rank in each repertoire: top 10, 11^th^–20^th^, 21^st^–40^th^, 41^st^–100^th^, and others. The total frequency of clones in each class is shown (n = 4). **(D)**, Clonality of the left and right CD4^+^ tumor repertoire (n = 4). **(E)**, Homoscedasticity plot for variance of clonality in individual mice. The X-axis represents the average clonality of bilateral tumors in each individual (n = 4). The Y-axis represents variance of clonality between different bilateral tumors within individuals. Sum of squares within mouse = 4.4 × 10^-4^, Sum of squares between mice = 1.3 × 10^-2^, Degree of freedom within mouse = 4, Degree of freedom between mice = 3.

Next, we investigated the equivalency of T cell clonal responses inside the bilateral tumors. CD4^+^ and CD8^+^ T cells from the tumor and CD4^+^ CD44^hi^ and CD8^+^ CD44^hi^ T cells from dLN were sorted 13 days after tumor inoculation, and their TCR repertoires were analyzed (**Figure 1C**: Experimental scheme for this study and **Supplementary Figure 1A**). Variable-Joining (V/J) segment usage of TCRβ, which is commonly used to characterize individual repertoire, was highly similar between bilateral tumors in the same mouse but varied among mice (**Figures 1D, E**). A heatmap depicting the relative proportion of each TRB V gene in each tumor repertoire showed that TRB V usage was highly similar between the bilateral tumors in the same mouse (**Supplementary Figure 2**). The proportion of the most abundant T cell clones was also similar between bilateral tumors, suggesting that they contained an equivalent number of expanded clones (**Figure 1F** and **Supplementary Figure 3**). Consistently, the clonality of the CD8^+^ T cell repertoire, which represents the extent of clonal expansion, was equivalent between the bilateral tumors (**Figure 1G**). In addition, the difference in clonality between the bilateral tumors of the same mouse was significantly smaller than that between different mice (**Figure 1H**). A similar trend was observed for the CD4^+^ T cell repertoire (**Figure 2**). These results demonstrated that the TCR repertoire features like V/J segment usage and clonality were similar between the bilateral tumors.

### The Majority of the T Cell Repertoire Was Composed of Shared Clones With a Similar Extent of Expansion in Bilateral Tumors

**Figure 3:**
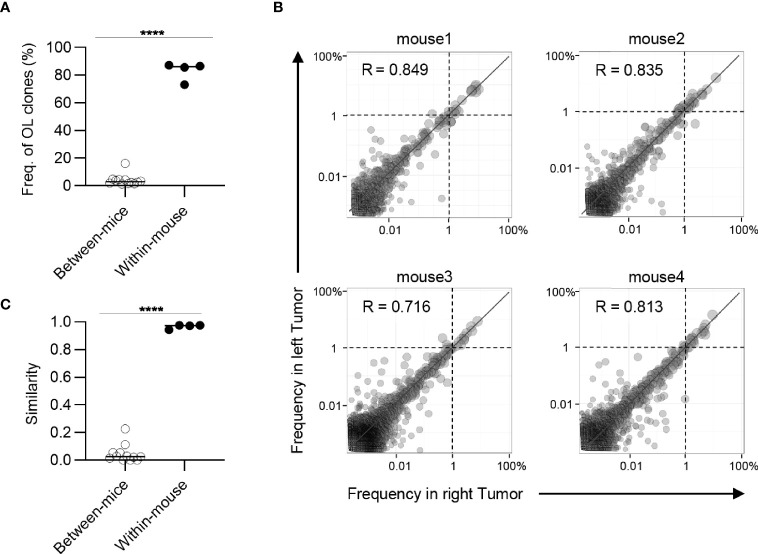
Clonal composition of CD8^+^ T cell repertoires in bilateral tumors. **(A)**, Comparison of the frequency of overlapping clones in tumors between and within mice (between mice, n = 4 × 3; within mice, n = 4). **(B)**, Scatter plot of CD8^+^ T cell clones from bilateral tumors (n = 4). Each dot represents a single clone with indicated frequency in the left (X-axis) and right tumors (Y-axis). The dotted line indicates a frequency of 1%. **(C)**, Comparison of the similarity of the tumor repertoires between and within mice (n = 4). Mean; Two-sided unpaired Student’s t-test **(A, C)**; ****P ≤ 0.0001.

Highly similar Indices of the TCR repertoire between bilateral tumors suggested that T cell clones that might present on one side also exist on the other side. To investigate this possibility, we analyzed the frequency of T cell clones that overlapped between the bilateral tumors or different individuals (**Figure 3A**). The clones overlapping bilateral tumors accounted for approximately 80% of the CD8^+^ T cell repertoire. In addition, a clonal overlap heat map for all tumors showed that the frequency of the clones that overlapped between the bilateral tumors was higher than the tumors from different individuals (**Supplementary Figure 4**). However, T cell clones overlapping between different mice covered only about 3%. This result indicated that most of the tumor-infiltrating CD8^+^ T cell clones were commonly present on both sides of the tumor within the same individuals.

**Figure 4:**
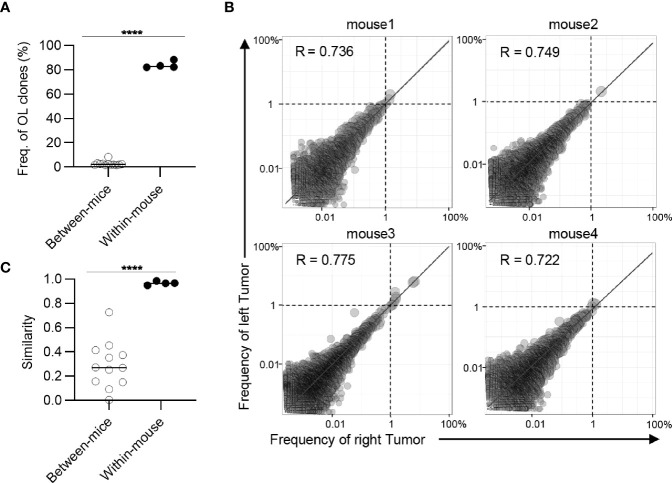
Clonal composition of CD4^+^ T cell repertoires in bilateral tumors. **(A)**, Comparison of the frequency of overlapping clones in tumors between and within mice (between mice, n = 4 × 3; within mice, n = 4). **(B)**, Scatter plot of CD4^+^ T cell clones from bilateral tumors (n = 4). Each dot represents a single clone with indicated frequency in the left (X-axis) and right tumors (Y-axis). The dotted line indicates a frequency of 1%. **(C)**, Comparison of the similarity of the tumor repertoires between and within mice (n = 4). Mean; Two-sided unpaired Student’s t-test **(A, C)**; ****P ≤ 0.0001.

Next, we examined whether these overlapping clones expanded equally in bilateral tumors. To this end, we depicted the frequency of each overlapping clone within the right (x-axis) and left (y-axis) tumors as a scatter plot (**Figure 3B**). The frequency of overlapping clones that had expanded to more than 1% was almost equal between the left and right sides. There were some clones whose frequency differed between the left and right sides, but the frequency of these clones was relatively low (**Supplementary Figure 5**). The number of overlapping clones was low between different mice, and the frequency of each clone was not correlated (**Supplementary Figure 6**). Consistent with this finding, the Morisita-Horn similarity index, which reflects the similarity of two T cell repertoires considering the frequency of each shared clone (29), was over 0.9 between the bilateral tumors, whereas it ranged from 0.05 to 0.3 among the tumors in different individuals (**Figure 3C**). The CD4^+^ T cell repertoire also showed similar tendencies (**Figure 4**). These results indicated that the majority of CD8^+^ T cell clones in tumors are shared between the bilateral tumors within the same individuals and these clones expanded to a similar extent, suggesting the clonal T cell responses on one side reflects those on the other side in our bilateral tumor model.

### The Similarity of TCR Repertoires Between Bilateral Tumors Was Equivalent to the Similarity Within the Tumor

**Figure 5:**
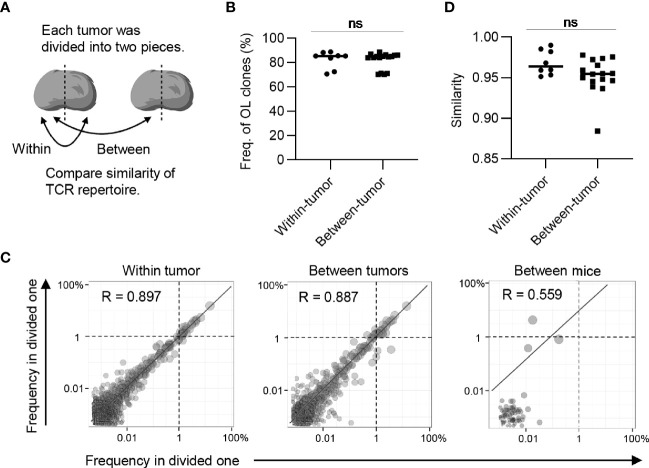
The extent of difference of CD8^+^ T cell repertoires between bilateral tumors. **(A)**, Schematic diagram of the analysis. **(B)**, Comparison of the frequency of the clones overlapped between divided same side tumors and bilateral tumor fragments (n = 4). **(C)**, Scatter plot of CD8^+^ T cell clones from bilateral tumors. Each dot represents a single clone with indicated frequency in each tumor fragment. The dotted line indicates a frequency of 1%. **(D)**, Comparison of the similarity of the tumor repertoires between divided same tumors and bilateral tumor fragments. **(B, D)** Within-tumor, n = 4 × 2; between-tumor, n = 4 × 4; Mean; Two-sided unpaired Student’s t-test; ns, non-significant.

**Figure 6:**
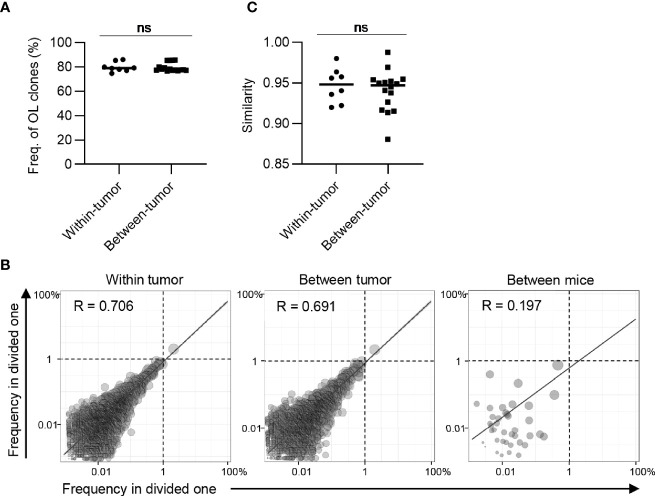
The extent of difference of CD4^+^ T cell repertoires between bilateral tumors. **(A)**, Comparison of the frequency of the clones overlapped between divided same side tumors and bilateral tumor fragments (n = 4). **(B)**, Scatter plot of CD4^+^ T cell clones from bilateral tumors. Each dot represents a single clone with indicated frequency in each tumor fragment. The dotted line indicates a frequency of 1%. **(C)**, Comparison of the similarity of the tumor repertoires between divided same tumors and bilateral tumor fragments. **(A, C)** Within-tumor, n = 4 × 2; between-tumor, n = 4 × 4; Mean; Two-sided unpaired Student’s t-test; ns, non-significant.

A previous study has shown that there is intratumoral heterogeneity of the TCR repertoire (30). When extrapolating our bilateral tumor model to a clinical situation, where antitumor immune responses are longitudinally monitored by tumor biopsy, it is important to determine whether the difference in TCR repertoire between the bilateral tumors could be considered equivalent to that within one side of the tumor. To investigate this possibility, we analyzed the intra-tumoral similarity of TCR repertoire between two pieces of tumor divided within each side and compared the similarity with inter-tumoral TCR similarity (**Figure 5A**). The total frequency of CD8^+^ overlapping clones between the left and right tumors was approximately 80%, which was equivalent to the frequency of overlapping clones within one side (**Figure 5B**). Additionally, scatter plots depicting the frequency of each overlapping clone showed that the variance in frequency of overlapping clones between bilateral tumors was equivalent to that within the tumor and significantly lower than those between different mice (**Figure 5C**). Consistently, there was no significant difference in the similarity of the TCR repertoire between the tumor and within the tumor (**Figure 5D**). A similar tendency was observed for the CD4^+^ T cell repertoire (**Figure 6**). These data demonstrated that the differences in TCR repertoire between the bilateral tumors were equivalent to the TCR heterogeneity within each tumor. This also suggested that our bilateral tumor experiments could be considered as an experimental model for temporal monitoring of TCR repertoire using sequential tumor biopsy.

### Proportional Infiltration of T Cell Clones Into Bilateral Tumors Contributed to the Similarity of TCR Repertoires

**Figure 7:**
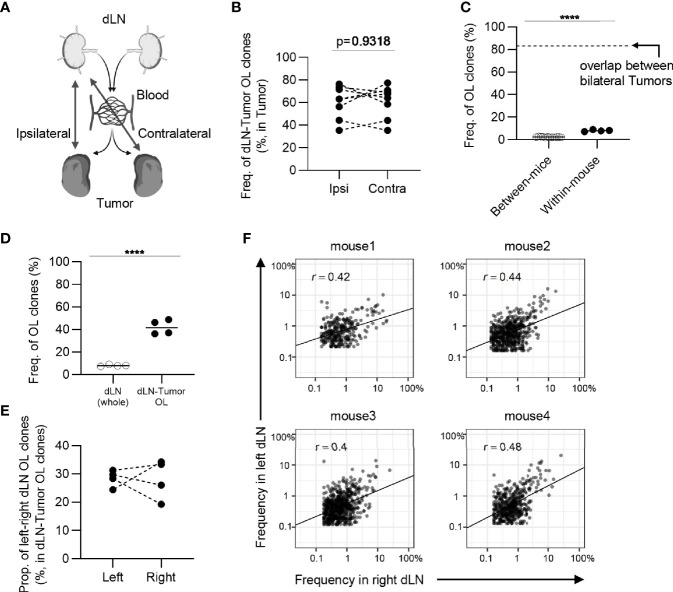
The similarity of CD8^+^ T cell repertoires in the bilateral dLNs. **(A)**, Schematic diagram of the hypothesis that T cell clones induced in dLN infiltrate into tumors evenly through blood circulation. **(B)**, The frequency of the clones overlapped between the dLN and its ipsilateral and contralateral tumor. Ipsi, ipsilateral; Contra, contralateral. n = 4 × 2. **(C)**, Comparison of the frequency of overlapping clones in dLN between and within mice (between mice, n = 4 × 3; within mice, n = 4). The dotted line indicates the frequency of clones overlapped between bilateral tumors within the mouse. **(D)**, Comparison of the frequency of left–right overlapping clones within whole dLNs and dLN-tumor overlapping repertoire (n = 4). **(E)**, The proportion of left–right overlapping clones within the dLN-tumor OL repertoires (n = 4). **(F)**, Scatter plot of left–right overlapping clones within the dLN-tumor OL repertoires (n = 4). Each dot represents a single clone with indicated frequency in each dLNs. Mean; Two-sided unpaired Student’s t-test; ns, non-significant. ****P ≤ 0.0001.

Tumor-reactive clones that expand in the bilateral dLNs exit from the efferent lymph, enter the blood circulation *via* the thoracic duct and eventually form a blood repertoire. Thus, we hypothesized that the tumor-reactive clones induced in one side of the dLNs infiltrate into the bilateral tumors proportionally through the circulation and then proliferate *in situ* at the same rate (**Figure 7A**). To investigate this idea, we examined whether the frequency of overlapping clones between the tumor and its contralateral dLN was equivalent to that of overlapping clones between the ipsilateral ones (**Figure 7B**). The total frequency of overlapping clones between contralateral dLNs and the tumor was almost the same as that between the ipsilateral ones, which supported our hypothesis.

**Figure 8:**
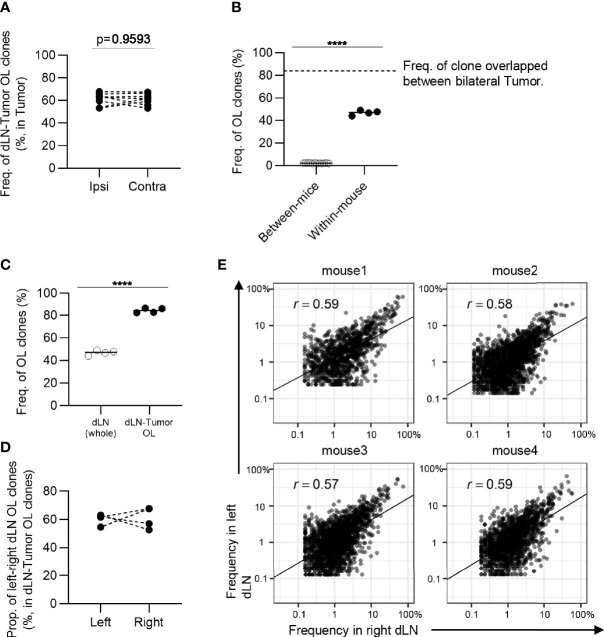
The similarity of CD4^+^ T cell repertoires in the bilateral dLNs. **(A)**, The frequency of the clones overlapped between the dLN and its ipsilateral and contralateral tumor. Ipsi, ipsilateral; Contra, contralateral. n = 4 × 2. **(B)**, Comparison of the frequency of overlapping clones in dLN between and within mice (between mice, n = 4 × 3; within mice, n = 4). The dotted line indicates the frequency of clones overlapped between bilateral tumors within the mouse. **(C)**, Comparison of the frequency of left–right overlapping clones within whole dLNs and dLN-tumor overlapping repertoire (n = 4). **(D)**, The proportion of left–right overlapping clones within the dLN-tumor OL repertoires (n = 4). **(E)**, Scatter plot of left–right overlapping clones within the dLN-tumor OL repertoires (n = 4). Each dot represents a single clone with indicated frequency in each dLNs. Mean; Two-sided unpaired Student’s t-test; ns, non-significant. ****P ≤ 0.0001.

However, it is unclear whether similar clones were induced in bilateral dLNs. To address this question, we examined the frequency of overlapping clones in the bilateral dLNs and found that it was substantially lower (7 to 10%) than the overlap between bilateral tumors (70 to 90%), although it was higher than the overlap between individuals (less than 3%) (**Figure 7C**). It was possible that dLN CD8^+^ CD44^hi^ T cells contained a substantial proportion of non-tumor-associated clones, resulting in the reduced overlap between bilateral dLNs. Therefore, we next examined the TCR repertoire of bilateral dLNs that overlapped with either of the bilateral tumors to enrich tumor-associated clones. In dLN-tumor overlapping clones in the bilateral dLNs, the frequency of left-right overlapping clones between bilateral dLNs increased to approximately 40% (**Figure 7D**). In terms of the number of clones, the proportion of left-right overlap was approximately 20 to 40% (**Figure 7E**). In other words, more than 50% of dLN-tumor overlapping clones were induced only in one of the dLNs. Finally, we investigated whether these dLN-tumor overlapping clones were equally expanded in the bilateral dLNs. We found that the correlation in frequency in dLN-tumor overlapping clones was moderate between the bilateral dLNs (0.4 ≤ r ≤ 0.48; **Figure 7F**), compared to the correlation between the bilateral tumors (0.71 ≤ r ≤ 0.85; **Figure 3B**). The frequency of CD4^+^ T cell clones overlapping between bilateral dLNs was higher than that of CD8^+^ T cell clones, and the CD4^+^ T cell repertoire also showed similar tendencies (**Figure 8**). A heatmap of the frequency of overlapping clones and Morisita Horn index of overlapping repertoires also showed that the similarity between the bilateral dLN repertoires in the same mouse was smaller than that between the bilateral tumors (**Supplementary Figure 7, 8**).

Collectively, these results suggested that the tumor-reactive T cell clones induced in the bilateral dLNs were only moderately conserved, and the degree of expansion of the shared clones differed between the bilateral dLNs. Therefore, the proportional infiltration of T cell clones into bilateral tumors through blood circulation contributed to the highly similar TCR repertoire of bilateral tumors.

## Discussion

The bilateral subcutaneous tumor model, where tumor cells were inoculated bilaterally into the backs of mice, is a promising model for temporal analysis of the antitumor response in cancer immunotherapy. In this study, we examined the prerequisite for this strategy: the TCR repertoire is conserved between bilateral tumors with similar growth rates. We found that bilateral tumors that grow symmetrically contained a highly similar CD8^+^ and CD4^+^ T cell repertoire in our experimental model. Interestingly, the TCR repertoires in bilateral dLNs were less conserved than those in bilateral tumors. These results suggest that T cell clones induced in the bilateral dLNs eventually mixed into a blood repertoire proportionally infiltrated the bilateral tumors, and proliferated *in situ* at a similar rate. These findings provide the basis for analyzing temporal and treatment-induced changes in tumor-reactive T cell clones using a bilateral tumor model in mice.

Previous reports have examined the relationship between the immune microenvironment before ICI treatment and the antitumor response in bilateral tumor models; Zemeck et al. reported that in bilateral tumor models, the transcriptional signature of tumors before ICI treatment was different between responders and non-responders (19). Chen and colleagues analyzed the bilateral tumor models treated with ICI and reported that tumor-infiltrating CD8^+^ T cells in non-responders had an exhaustion signature while responders had an activation signature (20). All of these reports suggest that similar antitumor immune responses are induced in bilateral tumors. Our finding of a highly similar TCR repertoire in bilateral tumors may explain why similar immune responses are induced bilaterally. It is also interesting whether the transcriptional profile of a particular clone is conserved in both tumors. Thus, we plan to combine the single-cell immune repertoire analysis and bilateral tumor model to answer this question in a future study.

Although we did not test other mouse tumor models, our bilateral tumor model would be applicable in other tumor models, because the conserved TCR repertoire between the bilateral tumors seems to be dependent on an anatomical mechanism that is conserved among individuals. Notably, the difference in tumor size may alter the tumor microenvironment, such as the concentrations of chemo-attractants and vascularity, and it may decrease the similarity in TCR repertoire between the bilateral tumors. Verifying the bilateral symmetry of tumor growth or therapeutic response is necessary to evaluate the temporal changes in the tumor-reactive T cell repertoire using this model.

In humans, patient background, such as cancer type and stage, and history of treatment are associated with prognosis (31). Thus, it is difficult to obtain a large cohort of homogeneous patients for statistical analysis. Moreover, temporal tumor biopsy in patients is highly invasive. Considering these difficulties, clinical studies to validate the relationship between TCR repertoire and antitumor effects are limited. We believe that our bilateral tumor mouse model will overcome these barriers and provide immunological bases for the development and evaluation of immunotherapeutic agents.

A possible application of this bilateral tumor model is to investigate the predictive and prognostic features of TCR repertoire in ICI therapy. Considering that the antitumor responses following ICI therapy vary even among mice with the same genetic background, differences in the clonal T cell responses may reflect the variance of antitumor immune responses among syngeneic mice. We plan to examine the hypothesis that a large amount of dLN-tumor repertoire overlap before treatment would predict a better therapeutic response to ICIs using the bilateral tumor model. Clinically, we observed that an increased frequency of tumor-blood overlapping clones in blood CD8^+^ T cells before treatment was associated with a favorable clinical response to PD-1 blockade in gastrointestinal cancer (32). In addition, the bilateral tumor model enables temporal tracking of endogenous T cell clones in the same tumor-bearing mouse with or without therapeutic intervention. We expect that temporal analysis of endogenous T cell clones using bilateral tumor model will reveal the kinetics of expansion, contraction, and exhaustion of individual T cell clones in tumor-bearing host.

Overall, this study reported the TCR repertoire analysis of bilateral tumor models, which enables the evaluation of temporal and treatment-induced changes in the tumor-reactive T cell clones. We believe that this novel experimental system will deepen our understanding of the clonal responses of tumor-reactive T cells and contribute to the development and evaluation of immunotherapeutic agents.

## Data Availability Statement

The datasets generated for this study can be found in the NCBI GEO; accession GSE174225.

## Ethics Statement

The animal study was reviewed and approved by Animal ethics committee of Tokyo university of science.

## Author Contributions

MT, HA, SS, KM, and SU designed research. MT, HA, and HS performed research. MT, HA, and SU analyzed data. MT, HA, and SU wrote the initial draft of the manuscript. All the authors participated in writing the final manuscript.

## Funding

This work was supported by the Japan Society for the Promotion of Science under Grant Number 20281832 and 17929397, and by the Japan Agency for Medical Research and Development (AMED) under Grant Number JP 21gm6210025. HA was supported by the Tadamitsu Kishimoto Fellowship Program.

## Conflict of Interest

HA reports stock for ImmunoGeneTeqs, Inc. SU reports advisory role for ImmunoGeneTeqs, Inc; stock for ImmunoGeneTeqs, Inc, IDAC Theranostics, Inc. SS reports advisory role for ImmunoGeneTeqs, Inc; stock for ImmunoGeneTeqs, Inc., KM reports consulting or advisory role for Kyowa-Hakko Kirin, ImmunoGeneTeqs, Inc; research funding from Kyowa-Hakko Kirin, and Ono; stock for ImmunoGeneTeqs, Inc, IDAC Theranostics, Inc.

The remaining authors declare that the research was conducted in the absence of any commercial or financial relationships that could be construed as a potential conflict of interest.

## Publisher’s Note

All claims expressed in this article are solely those of the authors and do not necessarily represent those of their affiliated organizations, or those of the publisher, the editors and the reviewers. Any product that may be evaluated in this article, or claim that may be made by its manufacturer, is not guaranteed or endorsed by the publisher.

## Supplementary Material

The Supplementary Material for this article can be found online at: https://www.frontiersin.org/articles/10.3389/fimmu.2021.744381/full#supplementary-material

Supplementary Figure 1 Gating strategy for CD4+ and CD8+ T cells in the tumor and CD4+CD44hi and CD8+CD44hi T cells in the draining lymph node (dLN).

Supplementary Figure 2 Heatmap depicting the relative proportion of each TRB V gene. Tumor samples were grouped by unsupervised hierarchical clustering based on the normalized values (Z values) of the relative proportion of each TRB V gene. Each column represents a TRB V gene, and each row represents a tumor sample (n = 4 × 2).

Supplementary Figure 3 Frequency of abundant T cell clones in the tumor and dLN. CD8+ and CD4+ T cell clones were categorized into five classes based on their rank in each repertoire: top 10, 11th–20th, 21st–40th, 41st–100th, and others. The total frequency of clones in each class is shown (n = 4).

Supplementary Figure 4 Clonal overlap heatmap for all tumors. Heatmap showing the geometric mean of frequency of overlapping clones between all sequenced tumor repertoire combinations (n = 4). Samples were grouped by unsupervised hierarchical clustering.

Supplementary Figure 5 Identification of T cell clones differentially expanded between bilateral tumors. A, Scatter plot of T cell clones from bilateral tumors. Each dot represents a single clone with indicated frequency in the right (X-axis) and the left tumors (Y-axis). Clones that are differentially expanded in one side of the tumor (differentially expanded clones) are shown in red or blue. The dotted line indicates a frequency of 1% (n = 4). B, Proportion of differentially expanded T cell clones (n = 4). Clones are categorized based on their averaged frequency in bilateral tumors (X-axis), the proportion of differentially expanded clones identified as (A) was calculated (Y-axis).

Supplementary Figure 6 Scatter plot of CD8^+^ and CD4^+^ T cell clones from different mice. Each dot represents a single clone with indicated frequency in each tumor (n = 4). Only the plot between the repertoire of mouse1_left and mouse2, 3, or 4_left was shown.

Supplementary Figure 7 The similarity of each TCR repertoire. Heatmap of the geometric mean of frequency of overlapping clones for all repertoires of divided tumor and dLN (n = 4). Samples were grouped by unsupervised hierarchical clustering.

Supplementary Figure 8 The similarity of each TCR repertoire. Heatmap of the Morisita-Horn index of overlapping clones for all repertoires of divided tumor and dLN (n = 4). Samples were grouped by unsupervised hierarchical clustering.
