# Supplementary material for: Proportional Tumor Infiltration of T Cells via Circulation Duplicates the T Cell Receptor Repertoire in a Bilateral Tumor Mouse Model
Source: Front Immunol. 2021 Oct 25;12:744381. doi: 10.3389/fimmu.2021.744381 (PMC8573377; doi:10.3389/fimmu.2021.744381)

Supplementary Figure 1.

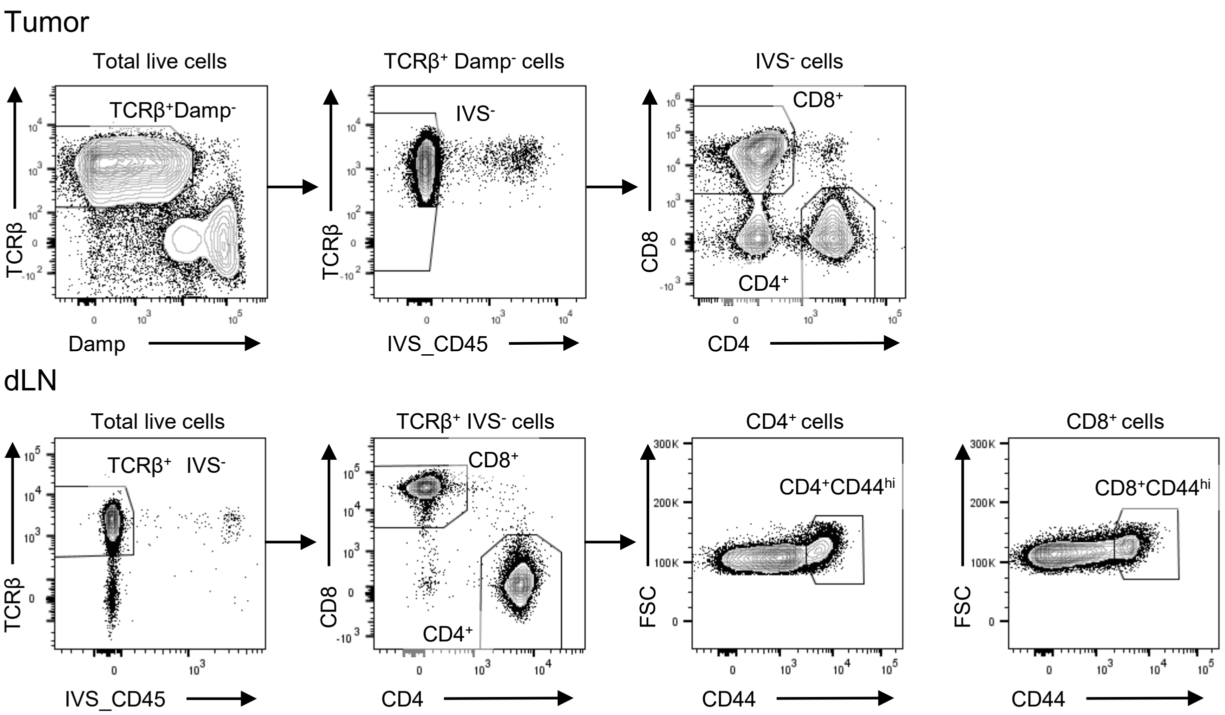

Supplementary Figure 2.

CD8+ TCR repertoire

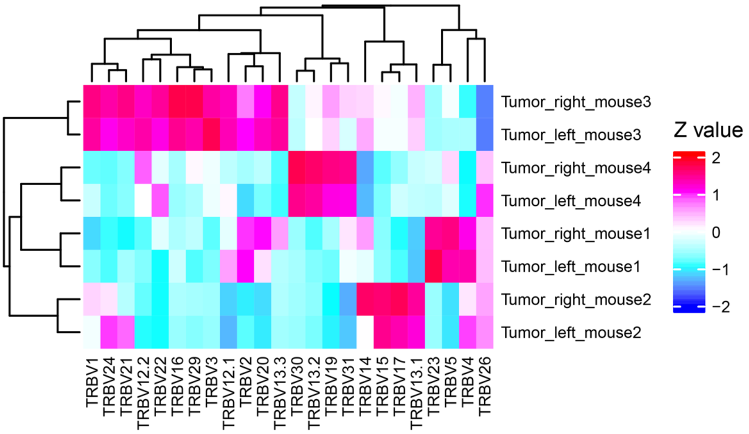

CD4+ TCR repertoire

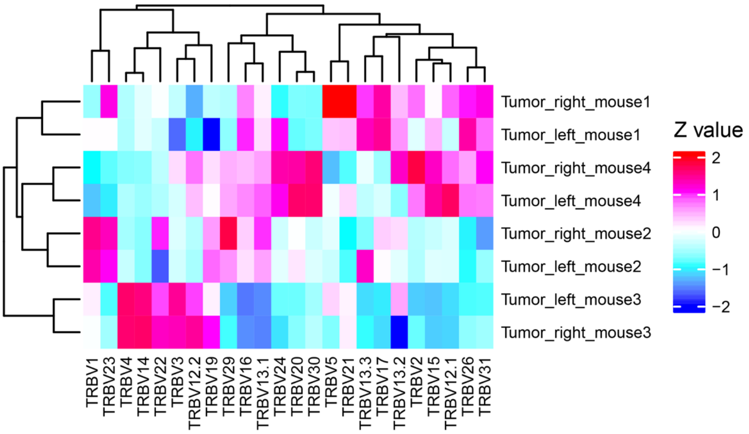

Supplementary Figure 3.

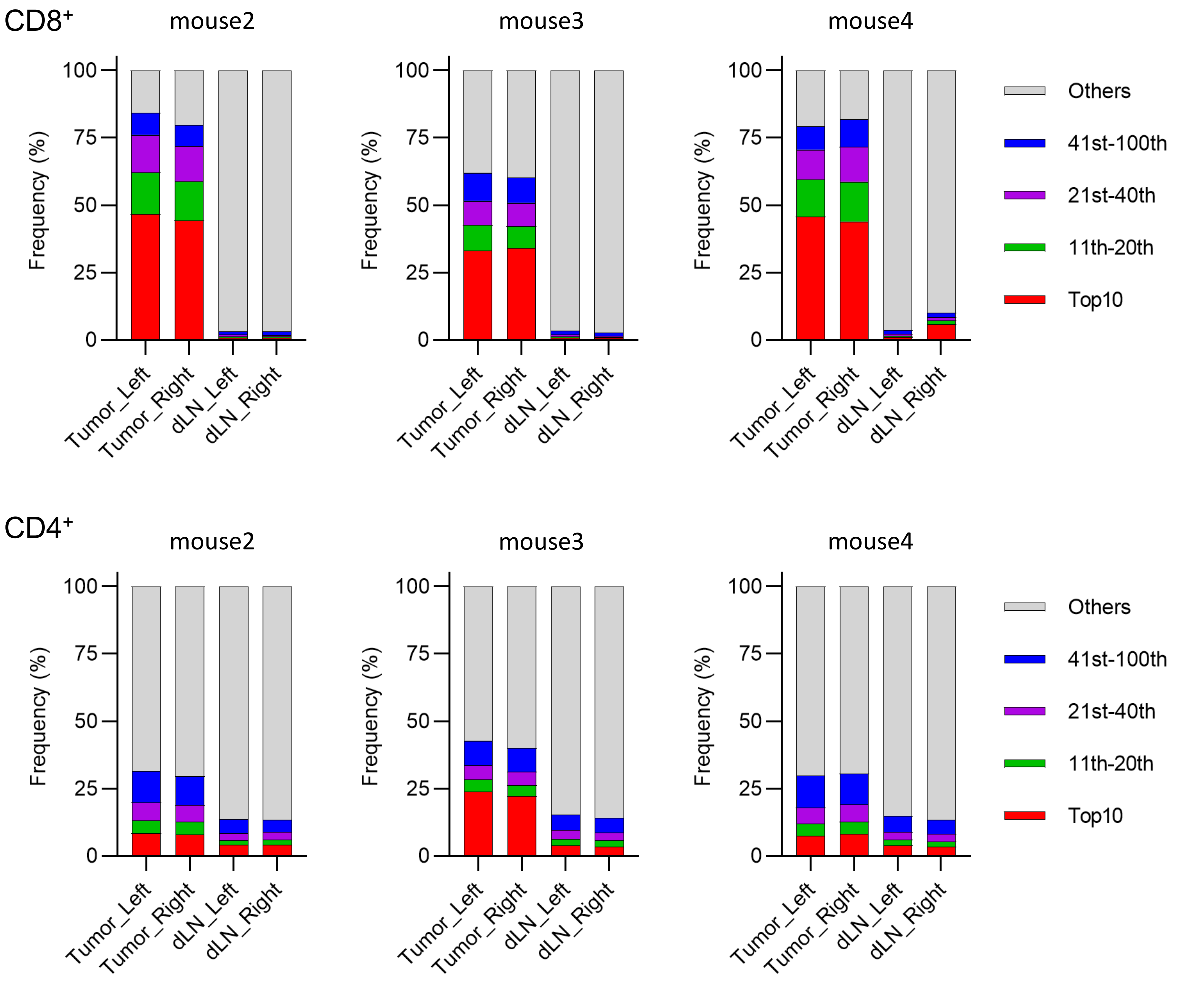

Supplementary Figure 4.

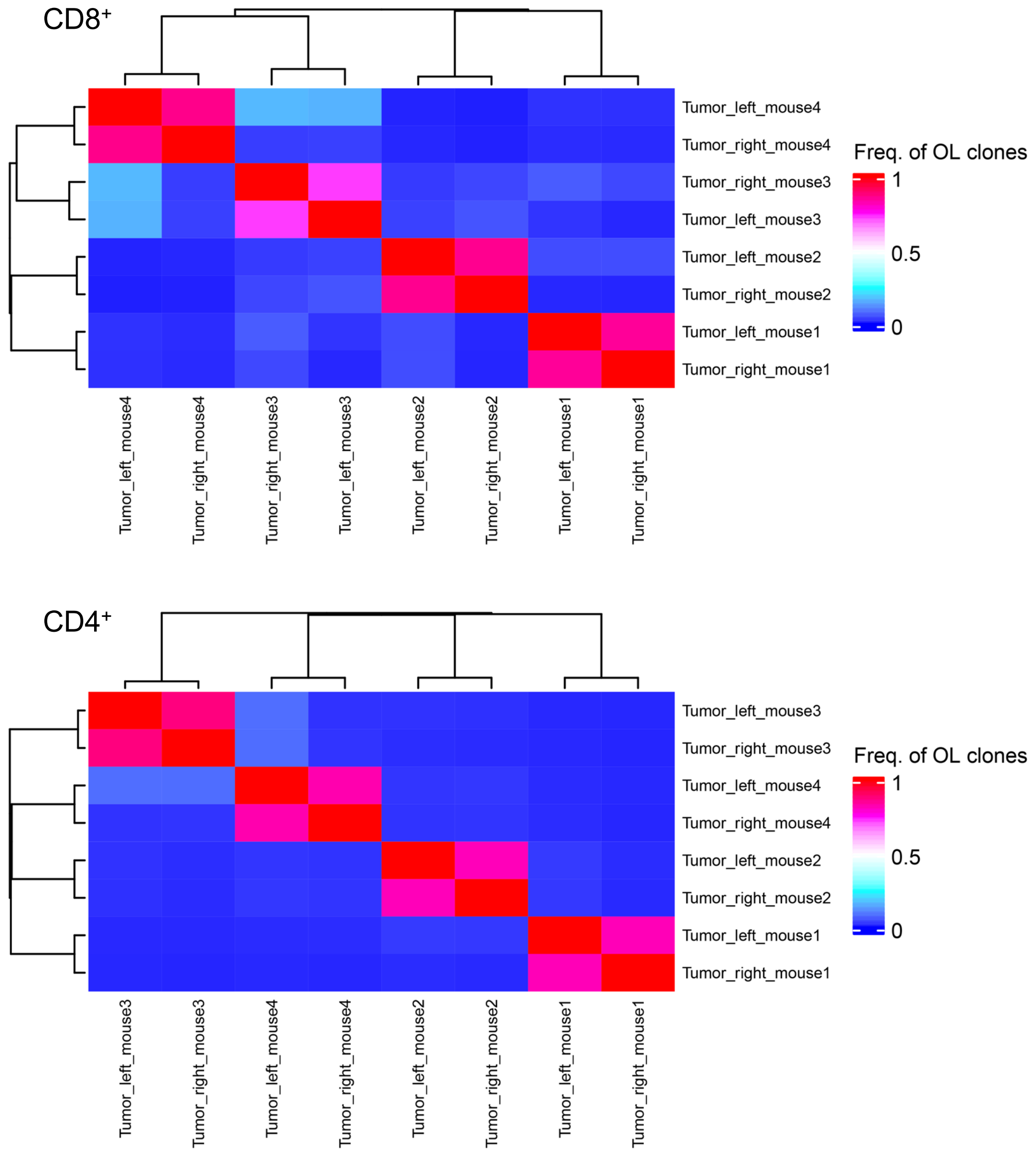

Supplementary Figure 5.

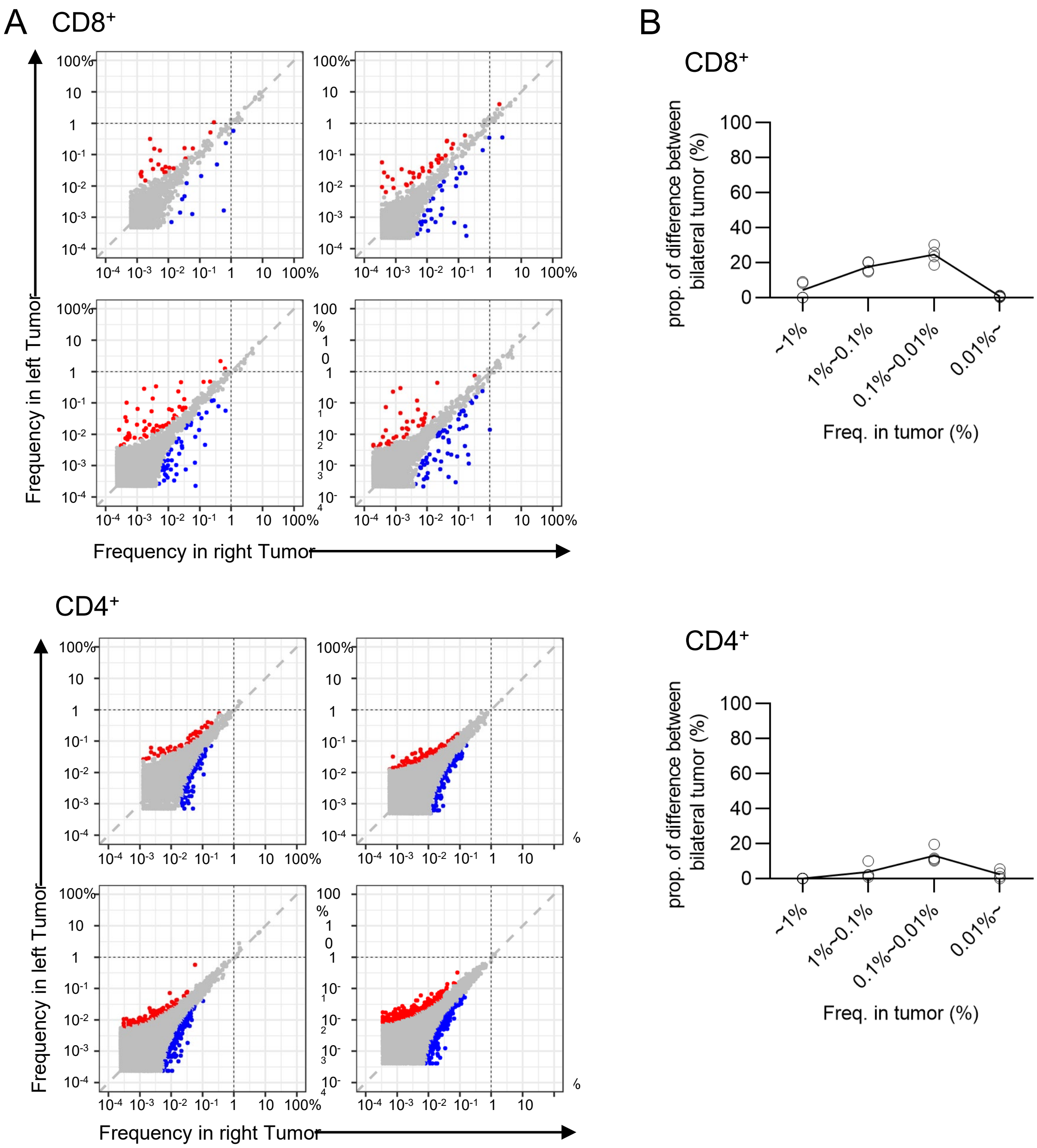

Supplementary Figure 6.

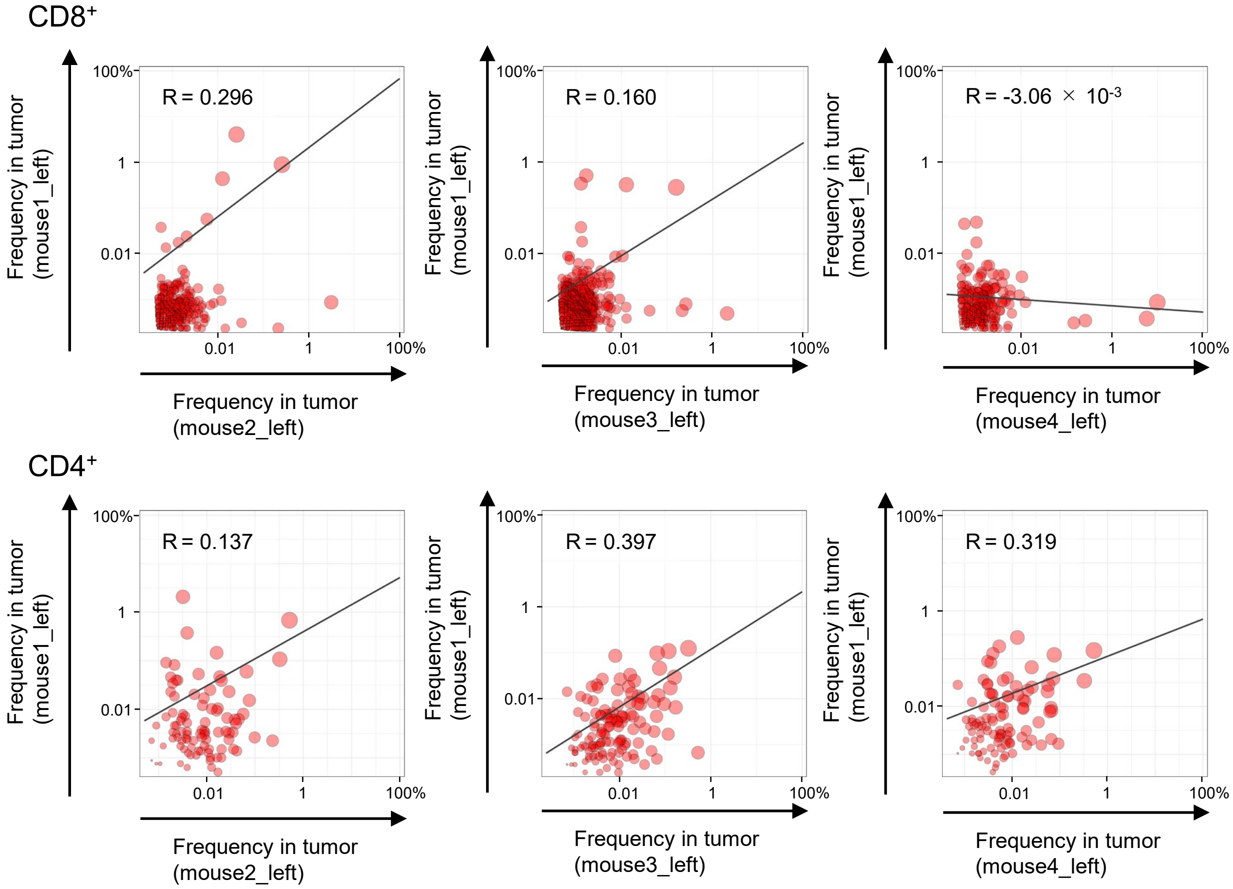

Supplementary Figure 7.

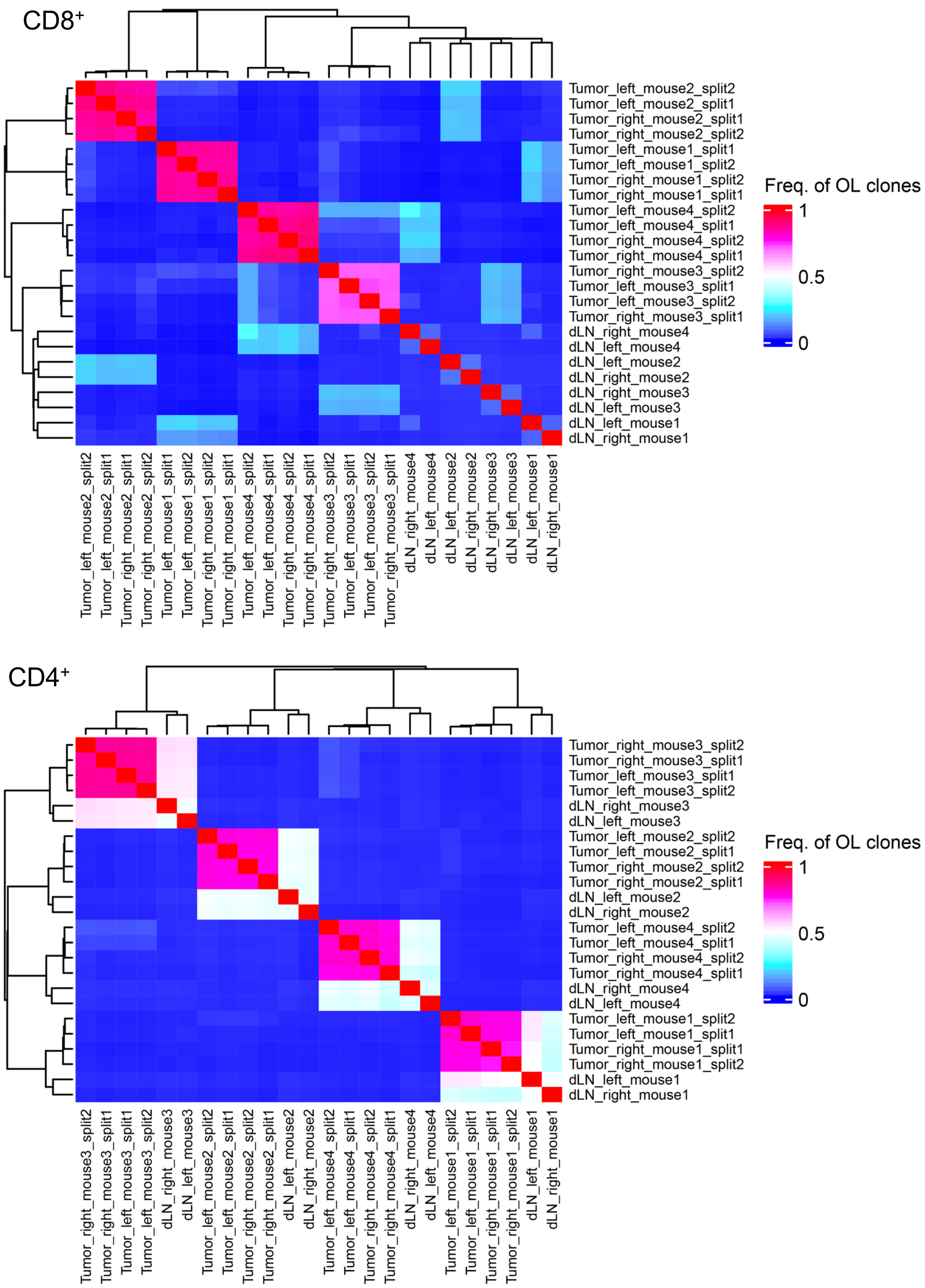

Supplementary Figure 8.

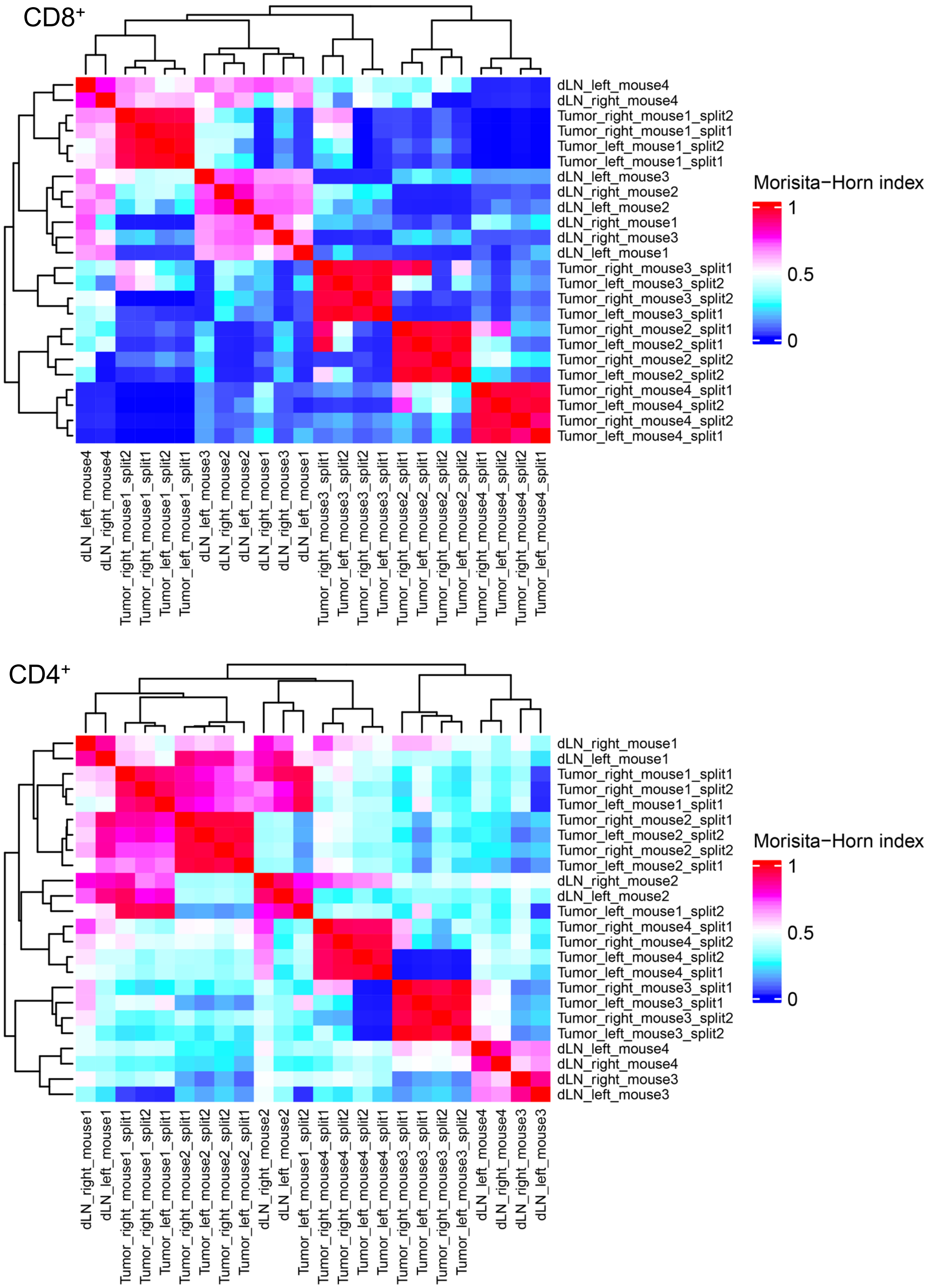

Supplement: Supplementary Figure 1 — Gating strategy for CD4+ and CD8+ T cells in the tumor and CD4+CD44hi and CD8+CD44hi T cells in the draining lymph node (dLN). [file DataSheet_1.pdf]
